# DNA methylation and adaptive response in forest tree species

**DOI:** 10.1186/1753-6561-5-S7-P86

**Published:** 2011-09-13

**Authors:** Angeles Guevara, Enrique Sáez, Luis-Manuel Díaz, Tamara Herranz, Carmen Barbero, Dolóres Vélez, Carmen Collada, David Sánchez-Gómez, Ismael Aranda, Teresa Cervera

**Affiliations:** 1 Dpto. Ecología y Genética Forestal, CIFOR-INIA, Ctra.de La Coruña km 7, 28040 Madrid, Spain; 2 Dpto. de Biotecnología. ETSIM (UPM), Ciudad Universitaria s/n, 28040 Madrid, Spain; 3 Dpto. Silvopasticultura. ETSIM (UPM), Ciudad Universitaria s/n, 28040 Madrid, Spain

## 

Progressive increase of temperatures as well as longer seasonal drought periods revealed by climate studies correspond to fast environmental changes that forest species face with their actual genetic background. Natural selective processes cannot develop an adaptive response within this time frame. Thus the capability of forest tree species to adapt to the new environments will depend on their genetic background, but also rely on their phenotypic plasticity. Several reports have shown the involvement of epigenetic modifiers as the basis of the phenotypic plasticity, and in particular to the adaptation to abiotic stresses. DNA methylation (methylation of cytosine residues)is one the most important epigenetic modification in eukaryotes. Itis involved in specific biological processes such as gene transcription regulation, gene silencing, mobile element control or genome imprinting.Therefore, there is a great interest in analyzing cytosine methylation levels and distribution within the genome.

In order to analyze methylation-sensitive anonymous CCGG restriction sites we used MSAP technique (Methylation-Sensitive Amplified Polymorphism), an AFLP-based technique for the analysis of cytosine methylation. The technique is based on the use of isoschizomers that show differential cleavage sensitivity to cytosine methylation. *Hpa*II and *Msp*I are isoschizomers that are frequently used to detect cytosine methylation. *Hpa*II cannot cleave if one or both cytosines are fully methylated (in both strands), whereas *Msp*I cleaves C^5m^CGG but not ^5m^CCGG sequences). For each sample, MSAP analysis is performed using both *Eco*RI/*Hpa*II and *Eco*RI/*Msp*I digested samples. Comparative analysis between *Eco*RI/*Hpa*II and *Eco*RI/*Msp*I fragment patterns allows the identification of two types of polymorphisms: (1) “Methylation-insensitive polymorphisms” that are associated with genetic variability and will show common *Eco*RI/*Hpa*II and *Eco*RI/*Msp*I profiles among samples; and (2) “Methylation-sensitive polymorphisms” that are associated with epigenetic variability and detected as amplified fragments differing in their presence or absence or in their intensity between *Eco*RI/*Hpa*II and *Eco*RI/*Msp*I patterns of the same sample. Thus, full methylation of the internal cytosine at the assayed CCGG sites will be associated with fragments detected in *Eco*RI/*Msp*I pattern which are absent or less intense in *Eco*RI/*Hpa*II profile. Hemi-methylation of the external cytosine will be associated with fragments observed in *Eco*RI/*Hpa*II pattern which are absent in *Eco*RI/*Msp*I profile.

We have optimized DNA methylation analysis for two forest tree species, including both angiosperm (*Fagus* sp.) and gymnosperm (*Pinus* sp.) species.

The set-up of the MSAP technology has allowed study intra-specific variability of DNA methylation in *Pinus pinea* L. a specie which is characterized by a low genetic variability and high level of phenotypic plasticity. Representative populations spanning the whole distribution of this specie within Spanish geography were chosen for this study, including coastal and interior populations. MSAP patterns from vegetative propagated trees were compared among and within populations. Percentage of methylation-sensitive polymorphisms was calculated and selective markers were identified.

To test a possible role of DNA methylation in the plasticity of *Fagus sylvatica* L. in response to water deficit we analyzed two populations of beech from Spain and Sweden which differ in the response to water stress. Patterns of DNA methylation clearly differed between populations. Although the rate of DNA methylation was similar between irrigated and non-irrigated trees in each polpulation after AMOVA, some specific MSAPs associated with water stress response were identified.

